# *Xanthomonas campestris* pv. *campestris*: Current Knowledge and Emerging Biological Control Strategies Using Phyllosphere- and Rhizosphere-Associated *Bacillus* spp.

**DOI:** 10.3390/pathogens15090974

**Published:** 2026-09-14

**Authors:** Aleksandra Jelušić, Tatjana Popović Milovanović

**Affiliations:** 1 University of Belgrade, Institute for Multidisciplinary Research, National Institute of the Republic of Serbia, Kneza Višeslava 1, 11030 Belgrade, Serbia; 2 Institute for Plant Protection and Environment, Teodora Drajzera 9, 11040 Belgrade, Serbia

**Keywords:** brassica crops, black rot, microbial antagonism, disease supression, regenerative agriculture

## Abstract

*Xanthomonas campestris* pv. *campestris*, the causal agent of black rot, is one of the most important bacterial pathogens affecting Brassicaceae crops worldwide. Its seed-borne nature, multiple routes of dissemination, and limited availability of resistant cultivars make black rot difficult to manage and underscore the importance of early and reliable pathogen detection. This review summarizes current knowledge on the epidemiology of *X. campestris* pv. *campestris*, with emphasis on advances in conventional and molecular approaches used for its detection and characterization, as well as on the currently used disease management strategies. Particular attention is given to biological control as a promising and sustainable alternative to conventional approaches. Recent studies on the use of *Bacillus* spp. for black rot control are discussed, with emphasis on their secondary metabolites and antimicrobial potential. Despite promising results including disease suppression, comparable to that achieved with chemical control, the translation of laboratory findings into reliable field applications remains challenging, particularly with regard to formulation and consistent efficacy under variable environmental conditions. Future research should focus on integrating current knowledge of pathogen epidemiology, diagnostics, and biological control to facilitate the development of sustainable and practically applicable strategies for black rot management in *Brassica* crops.

## 1. Introduction

*Brassicaceae* (crucifers) comprise a diverse group of plants of agricultural, economic, and nutritional importance that are cultivated worldwide as vegetables, oilseeds, fodder, organic fertilizers, and are highly appreciated for their rich content of vitamins, antioxidants, and other health-promoting bioactive compounds [1]. However, their yield and quality are severely threatened by numerous pests and pathogens, among which black rot, caused by plant pathogenic bacterium *Xanthomonas campestris* pv. *campestris* (Pammel) Dowson is regarded as one of the most destructive diseases of crucifers worldwide. The disease can raise yield losses up to 50% and 70% in susceptible cultivars of cabbage and cauliflower, respectively [2,3]. According to the Centre for Agriculture and Bioscience International (CABI), *X. campestris* pv. *campestris* is currently present in 93 countries across six continents, underscoring its global economic and phytosanitary importance [4]. European and Mediterranean Plant Protection Organization (EPPO) database shows that this pathogen has been classified on the A2 list (listed pests are present but not widely distributed and are subject to official control) in Jordan since 2013, while since 2018 it has been included among quarantine pathogens in Mexico and on the A1 list (listed pests are absent from the EPPO region) in Brazil (https://gd.eppo.int/taxon/XANTCA/categorization) (accessed on 10 September 2026).

A multitude of different prevention and suppression strategies (cultural, physical, chemical, and biological) have been adopted to control *X. campestris* pv. *campestris* [5]. Currently, copper (Cu) and Cu-based products are considered the main chemical control agents of black rot disease [6]. However, their excessive application in disease control not only has negative consequences on human health and the environment (soil health, water quality, and biodiversity in agricultural ecosystems), but also contributes to the selection of resistant pathogens [7,8]. Considering the negative effects of the excessive and prolonged use of chemical pesticides on human health and the environment, there is an increasing need to prioritize biological control measures as a part of integrated pest management (IPM). In line with global policies promoting environmentally responsible agriculture, increasing attention is being paid to the application of biopesticides and regenerative agriculture approaches. Worldwide, increasing environmental concerns, stricter regulations, consumer demand for safer food, and global sustainability initiatives, are accelerating the development and commercialization of biological control agents and biopesticides. Consequently, regenerative agriculture, which focuses on improving soil health, enhancing biodiversity, reducing synthetic chemical inputs, and strengthening beneficial plant–microbe interactions, is becoming increasingly recognized as a sustainable framework for crop production systems.

Use of beneficial microorganisms, such as bacteria *Bacillus* spp. (*B. cereus*, *B. thuringiensis*, *B. velezensis*) and *Pseudomonas* spp. (*P. aeruginosa*, *P. orientalis*, *P. synxantha*), their bioactive metabolites, and other environmentally friendly approaches for black rot disease management on various host plants is presented in numerous studies [6,9,10,11,12,13]. Here, the lifestyle and epidemiology of *X. campestris* pv. *campestris*, with particular emphasis on strategies used for management of black rot disease. Special attention is given to the potential of biological control approaches, especially the use of *Bacillus* spp. strains, exploitation of autochthonous (phyllosphere and rhizosphere) strains, and their colonization and persistence within different plant niches, highlighting their potential for use in environmentally friendly and sustainable disease management strategies.

## 2. Historical Review

Plant pathogenic bacterium *X. campestris* pv. *campestris* was identified by Pammel [14,15] on turnips in Iowa (USA) in 1892, when it was described as *Bacillus campestris*. Following the initial report, *X. campestris* pv. *campestris* was subsequently recorded in all major regions where cruciferous crops are cultivated. In 1939, based on a comprehensive examination of the cultural and biochemical characteristics of numerous Gram-negative plant pathogenic bacteria, Dowson proposed the recognition of three genera—*Bacterium*, *Pseudomonas*, and *Xanthomonas* [16]. The genus *Xanthomonas* included non-spore-forming, rod-shaped, Gram-negative bacteria possessing a single polar flagellum, although biflagellate or non-motile strains were occasionally observed, and characterized by the production of large, yellow, mucoid colonies on nutrient agar. According to this classification, *B. campestris* was reclassified as *X. campestris*. Although the taxonomy of the genus has since undergone extensive revisions, the designation *X. campestris* pv. *campestris* has been retained for the black rot pathogen of Brassicaceae. Within this pathovar, isolates are further differentiated into physiological races based on their compatible or incompatible interactions with differential *Brassica* cultivars [17,18]. To date, a total of 11 physiological races (designated as 1–11) have been described for *X. campestris* pv. *campestris* [18,19]. Initially, *X. campestris* pv. *campestris* was classified into five races (0–4) according to its interactions with differential *Brassica* cultivars, including *B. rapa* (cvs. Just Right Turnip, Tokyo Turnip Hybrid, and Seven Top Turnip) and *B. juncea* (Florida Broad Leaf India Mustard), while *B. oleracea* cv. Early Jersey Wakefield was used as a susceptible control [20]. Subsequently, Vicente et al. [17], following the analysis of a larger collection of *X. campestris* pv. *campestris* strains, expanded the classification to include six races (1–6). Further studies led to the identification of three additional races (7, 8, and 9) [21,22,23], while Cruz et al. [18] later described two novel races, designated races 10 and 11, from Portugal. Worldwide, races 1 and 4 are the most prevalent and virulent, whereas races 2, 3, 5, and 6 occur only rarely [19]. Race specificity is determined by the interaction between bacterial effector proteins and their corresponding plant immune receptors. However, the contribution of type III effectors to race specificity in *X. campestris* pv. *campestris* remains incompletely understood. Castañeda et al. [24] demonstrated that, among eight putative *avr* genes investigated, only *avrXccFM* affected race specificity, whereas deletion of the other seven avr genes did not alter pathogenicity. Horizontal gene transfer has also contributed to the genomic diversification of *X. campestris*, with laterally transferred genomic islands containing genes associated with pathogenicity and primary metabolism [25].

Race specificity may be influenced by interactions between bacterial effector proteins and corresponding plant immune receptors; however, the contribution of type III effectors to race specificity in *X. campestris* pv. *campestris* remains incompletely understood [25]. Castañeda et al. [24] demonstrated that, among eight putative *avr* genes investigated, only *avrXccFM* affected race specificity, whereas deletion of the other seven *avr* genes did not alter pathogenicity.

## 3. Symptoms and Impact

The earliest manifestations of infection in seedlings and transplants are frequently difficult to recognize. Cotyledons may initially display minor yellow discoloration and necrotic areas, but in many cases they deteriorate rapidly, shrivel, and detach from the plant before symptoms are observed. Lesions characteristic of the disease may form on emerging leaves, yet plants are often transplanted before these signs become apparent [17,26,27].

**Figure 1:**
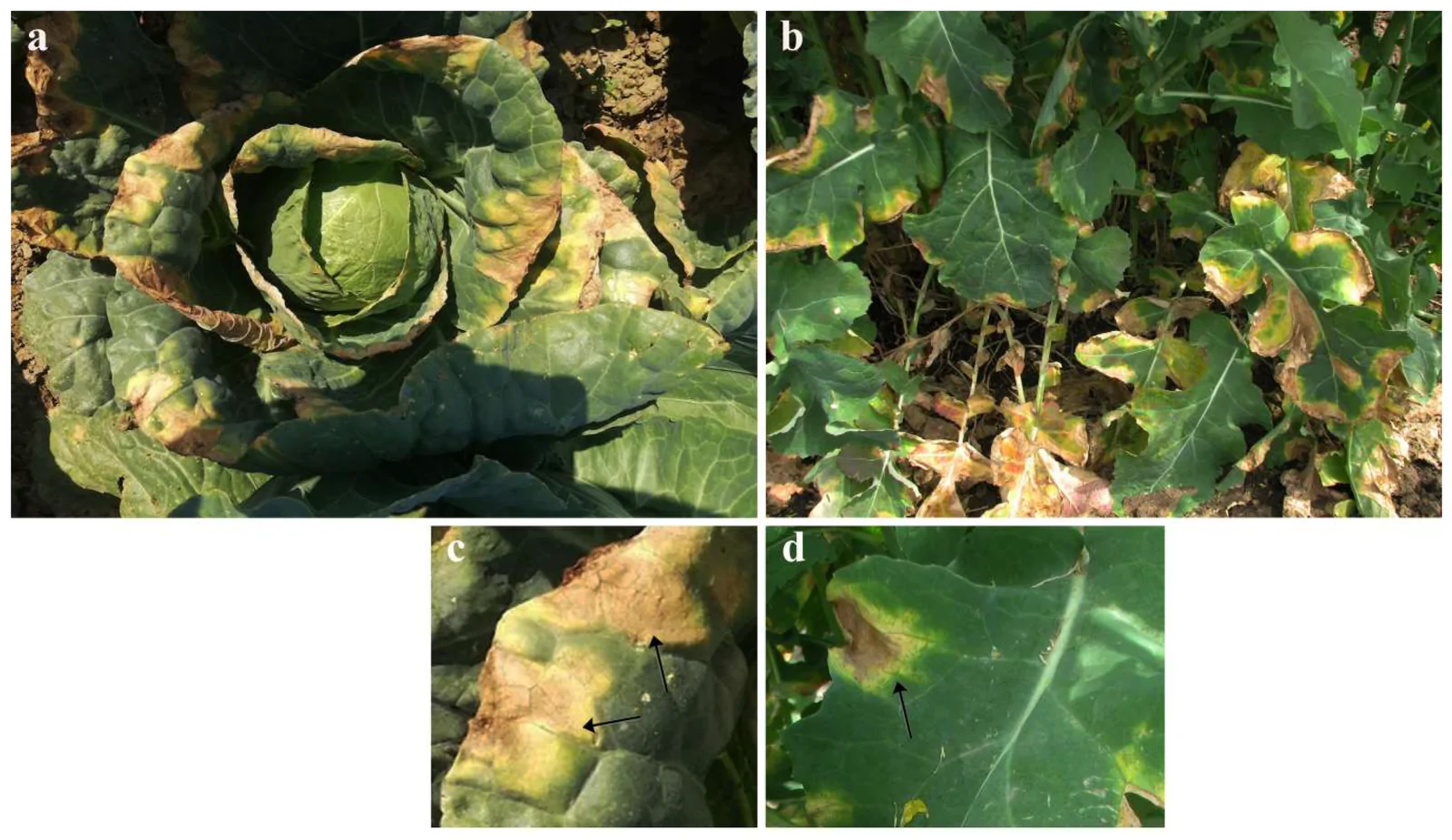
Symptoms caused by *Xanthomonas campestris* pv. *campestris* in naturally infected (**a**) cabbage and (**b**) oilseed rape plants. Close-up views showing characteristic V-shaped chlorotic to necrotic lesions extending inward from the leaf margins, with typical blackening of the veins, in cabbage (**c**) and oilseed rape (**d**) (arrows indicate the lesions and affected veins).

In field conditions, the most distinctive symptoms of the disease are V-shaped chlorotic to necrotic lesions that usually originate from the leaf margins and extend inward along the vascular tissues, often accompanied by darkening of the veins (Figure 1a–d). As the infection progresses, these lesions expand, become necrotic, and gradually turn pale brown and desiccated as a consequence of vascular tissue disruption and impaired water transport. Systemic colonization of the plant can be recognized by the discoloration of vascular bundles within the petiole and stem, frequently associated with unilateral or generalized wilting symptoms [26]. Under severe disease pressure, infected plants may exhibit reduced growth, developmental abnormalities, or complete plant death [17,21,28].

Premature shedding of infected leaves is a common consequence of disease development and may occur even when infection intensity is relatively low. This symptom is particularly significant in winter cauliflower production, where the loss of protective outer leaves exposes the curd, reducing its commercial value and making it unsuitable for market. In addition, secondary infections by soft-rot pathogens may develop, further intensifying tissue degradation and symptom severity in cabbage and cauliflower [17,27,28,29].

In some cases, disease expression may differ from the typical symptom pattern, appearing as dark, water-soaked lesions or larger necrotic areas on leaves. These infections can subsequently spread systemically, leading either to the characteristic vascular symptoms described above or to blight-like symptoms associated with tissue collapse and drying [21,27,30,31,32].

Typical symptoms of black rot disease are V-shaped chlorotic to necrotic lesions with blackened veins starting from the leaf margins and expanding as the pathogen migrates toward the leaf midvein (Figure 1a). This symptom pattern is characteristic of black rot in leafy *Brassica* vegetables. However, as reported by Jelušić et al. [31] and Cesbron et al. [33], infection of oilseed rape with *X. campestris* pv. *campestris* results in lesions resembling bacterial blight rather than the classical black rot symptoms, and are therefore described as bacterial blight-like symptoms (Figure 1b,d).

The ability of *X. campestris* pv. *campestris* to cause disease is supported by a complex set of virulence factors that promote host colonization, tissue invasion, and nutrient acquisition. These include exopolysaccharides (EPS), extracellular degradative enzymes (e.g., cellulases, mannanases, pectinases, and proteases), adhesins, toxins, lipopolysaccharides, and the Type III Secretion System (T3SS), a specialized apparatus responsible for delivering effector proteins into plant cells and modulating host defense responses [34]. In addition, the pathogen possesses two Type II Secretion Systems (T2SS), Xps-T2SS and Xcs-T2SS, which mediate the secretion of extracellular enzymes involved in plant cell-wall degradation and host tissue invasion. The Xps-T2SS is considered particularly important for virulence, as it facilitates the release of degradative enzymes that promote host colonization [35]. A critical component of *X. campestris* pv. *campestris* pathogenicity is cell-to-cell communication mediated by the diffusible signaling factor (DSF; cis-11-methyl-2-dodecenoic acid), which regulates the production of extracellular enzymes and polysaccharides, thereby facilitating host colonization and systemic spread through the vascular system [35]. The effectiveness of the mentioned pathogenicity mechanisms of *X. campestris* pv. *campestris*, together with its remarkable adaptability, contributes to its extensive distribution, broad host range, and substantial pathogenic variability. This variability is manifested through the existence of multiple physiological races, exhibiting differential pathogenicity toward various cruciferous crops that represents a major challenge for the effective disease management. Moreover, the considerable genetic diversity of *X. campestris* pv. *campestris* enables rapid adaptation to diverse environmental conditions and facilitates the development of resistance to control measures through the selection of resistant variants within the population and horizontal gene transfer [36]. Consequently, despite recent advances in conventional and molecular breeding aimed at introducing resistance genes, durable and broad-spectrum resistance remains difficult to achieve, as these genes often fail to provide effective protection against the diverse range of *X. campestris* pv. *campestri* isolates [37].

## 4. Epidemiology

*X. campestris* pv. *campestris*, the causal agent of black rot disease of cruciferous crops, is a seedborne pathogen causing substantial yield and quality losses, mainly in warm and humid climates [34,38]. Black rot represents a serious threat in leafy cruciferous vegetables cultivated for human consumption. In contrast, for crops in the oleifera and napobrassica groups, in which the harvested organs are seeds or roots, black rot generally has a less pronounced effect on final marketable yield. Nevertheless, the disease remains an important challenge during the early stages of their development. Infected seedlings often exhibit reduced growth, irregular development, and unilateral wilting or distortion of leaves. The infection at the seedling stage has been reported to reduce the plant’s photosynthetic ability and overall biomass resulting in the reduced crop yield [39]. Tissues colonized by *X. campestris* pv. *campestris* provide entry points for secondary fungal and bacterial pathogens, which can further reduce seed and root production [38].

**Figure 2:**
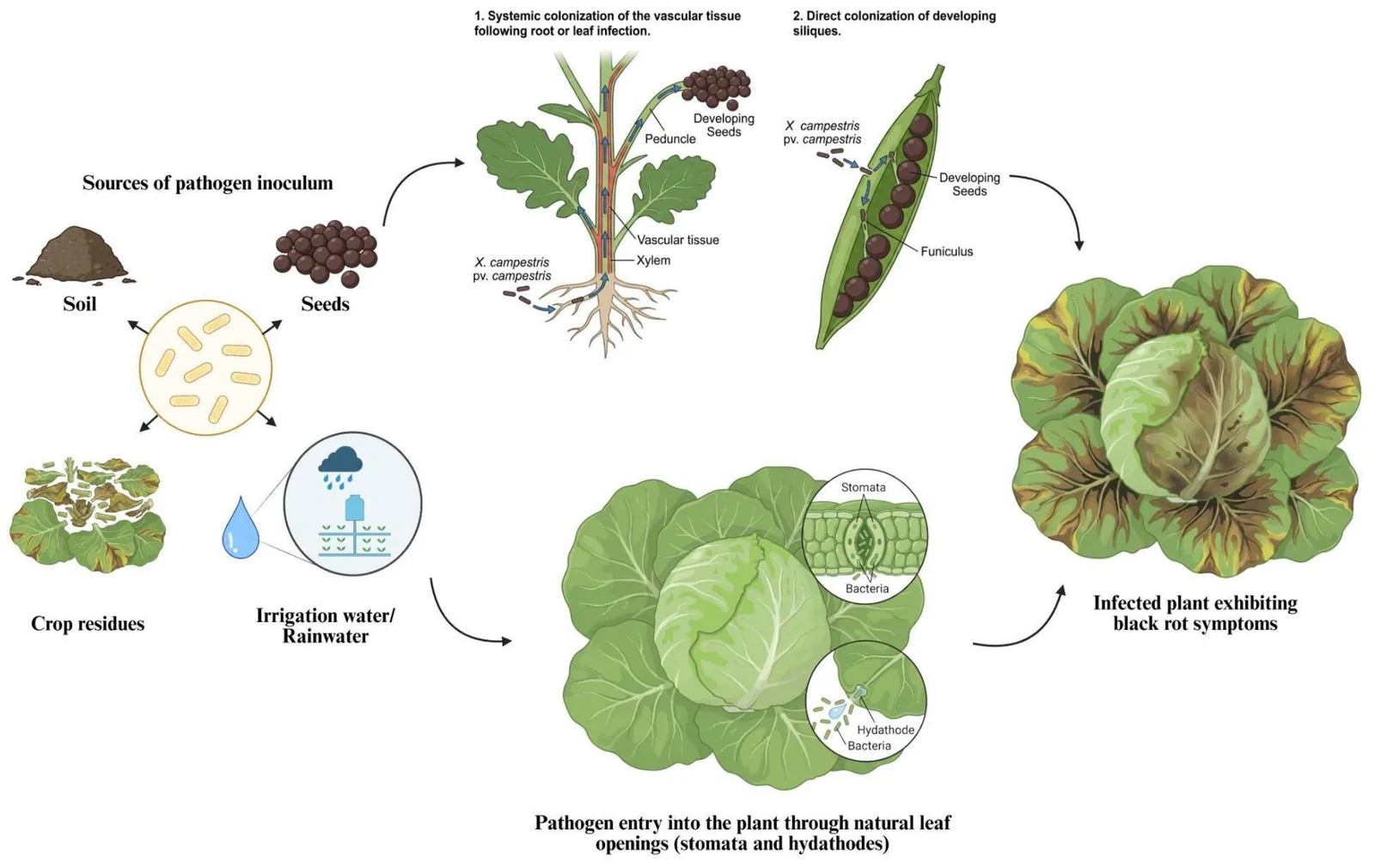
Life cycle of *Xanthomonas campestris* pv. *campestris* (created in https://BioRender.com); graphical representation of the information presented in this section.

Seeds serve as a primary source of pathogen inoculum, while alternative sources of infection are water splashing, aerosols dispersed from nearby infected fields, insects, human activity. (Figure 2) [40]. Van der Wolf et al. [41] further suggested that bacterial entry through stomata or wounds, followed by systemic movement through the vascular tissues of developing siliques, may provide a route for pathogen translocation into seeds. According to Vicente & Holub [19] the exact mechanism by which seeds become infected is not completely understood, although two potential routes are proposed. The first involves systemic colonization of the vascular tissue following root or leaf infection, allowing *X. campestris* pv. *campestris* to move upward through the xylem and potentially reach developing seeds via the peduncles; however, this pathway has not been conclusively demonstrated. The second, more widely accepted route involves direct colonization of developing siliques, from which the bacterium spreads through the funiculus to the developing seeds, resulting in seed contamination (Figure 2) [19,41]. Seed infection may involve pollen-mediated transmission. According to Dutta et al. [42], pepper blossoms may serve as a potential site of ingress for *Xanthomonas euvesicatoria* into developing seeds. The authors speculated that pollen germ tubes could potentially act as conduits for bacterial movement from colonized blossoms to developing ovules, although this mechanism was not experimentally confirmed. However, interestingly, the same study proved that *Acidovorax citrulli*, a nonhost pathogen of pepper, colonized pepper blossoms more effectively than *X. euvesicatoria*, suggesting that blossom-mediated seed infestation may be nonspecific and not strictly dependent on host–pathogen compatibility. Although pollen tube-mediated transfer has not been demonstrated for *X. campestris* pv. *campestris*, deep-seated infections of the endosperm and embryo have been demonstrated following flower inoculation with *X. campestris* pv. *campestris* [41].

The pathogen infects plants through natural openings on leaves, such as hydathodes and stomata, or mechanically generated wounds in the leaves and/or roots (Figure 2). According to An et al. [43], the infection cycle of *X. campestris* pv. *campestris* can be divided into two phases: (i) the epiphytic phase, which begins when the bacterium reaches the leaf surface of the host plant and lasts until its penetration into host tissues, and (ii) the endophytic phase, which starts once the bacterium enters and colonizes the host plant. To attach to the leaf surface, *Xanthomonas* species employ various adhesion strategies, including bacterial surface polysaccharides, adhesin proteins, and type IV pili, while their long-term persistence is facilitated by the formation of biofilm-like structures embedded in an extracellular polymeric matrix, which in *Xanthomonas* is mainly composed of xanthan and protects the bacteria from harsh abiotic stress conditions in the phyllosphere [43,44]. Their epiphytic survival is favored by moderately high temperatures (25–35 °C), which stimulate bacterial multiplication, while high humidity and rainfall provide favorable conditions for bacterial survival and facilitate access to natural openings such as stomata and hydathodes, thereby facilitating initial infection and pathogen spread [43,45,46]. Under favorable environmental conditions, epiphytically surviving bacterial cells may be dispersed to neighboring plants by rain splash or wind-driven water, thereby initiating new infection cycles [47,48]. Infection may be facilitated during the early morning hours, when guttation droplets are exuded from hydathodes at the leaf margins. Insects visiting or resting on leaf surfaces, as well as pollinating insects, may potentially contribute to the dissemination and transmission of *X. campestris* pv. *campestris*. Indeed, *X. campestris* pv. *campestris*-contaminated blue bottle flies (*Calliphora vomitoria*) have been shown to transmit the pathogen to cauliflower blossoms, resulting in seed infestation [40]. Similarly, Kastelein et al. [49] observed transmission of *X. campestris* pv. *campestris* between plots in polytunnel experiments where honeybees (*Apis mellifera*) were used for pollination; however, a direct role of the bees in pathogen transmission could not be confirmed.

Key strategies enabling epiphytic survival of *Xanthomonas* spp. are the production of the yellow membrane-bound pigment xanthomonadin, which protects bacterial cells from intense solar radiation (UV-A and UV-B) that can damage DNA, membranes, and proteins, as well as the production of extracellular polysaccharides (xanthan) or lipopolysaccharides which enhance tolerance to environmental stresses, promote biofilm formation, and facilitate both epiphytic survival and in plant colonization [43,48].

Upon entering the host plant, *X. campestris* pv. *campestris* initiates its endophytic phase and spreads systemically through the xylem vessels, which may result in characteristic vascular blackening associated with the accumulation of plant-derived melanin [36]. However, the occurrence and severity of vascular blackening are strain-dependent and are not necessarily observed in all infections, particularly during early stages or when strains induce blight rather than typical black rot symptoms [50]. During multiplication, the pathogen produces a large amount of xanthan which, together with bacterial cells, can obstruct xylem vessels and interfere with water transport. Vascular colonization and movement of the pathogen are associated with the development of characteristic V-shaped chlorotic to necrotic lesions originating at the leaf margins and extending toward the midrib, accompanied by blackening of the veins. (Figure 2) [2,3,51]. High temperature and humidity create favourable conditions for the pathogen multiplication, leading to systemic infection (wilting and rot) and ultimately causing considerable yield and economic losses [3]. *X. campestris* pv. *campestris* is mainly transmitted via contaminated seeds, but infected soil, crop residues, and irrigation or rainwater may also serve as sources of inoculum [3,37]. It has been demonstrated that, independently of the host plant, this bacterium can survive in soil for 20 to 40 days depending on the season (summer/winter), and up to two years when associated with plant debris in the soil [43]. According to Singh [52], soil-borne infection through roots is possible but its epidemiological significance remains unclear. Recent observations using fluorescence in situ hybridization and confocal microscopy confirmed the presence of *X. campestris* pv. *campestris* in roots following seed inoculation and demonstrated that pathogen incidence in roots does not necessarily correspond to the extent of colonization in stems [53]. Root injuries caused by soil particles or associated with lateral root emergence may provide potential entry points for the pathogen; however, the importance of these sites in *X. campestris* pv. *campestris* infection remains poorly characterized. Root hairs may also represent potential sites for bacterial entry or colonization, as reported for endophytic bacteria in other plant–bacterium interactions [54]. In addition, numerous wild cruciferous weeds can harbor *X. campestris* pv. *campestris* without obvious symptoms, acting as important reservoirs of the pathogen between growing seasons and contributing to pathogen dissemination [55]. However, weed-associated isolates are often genetically distinct from crop isolates, and their ability to cause disease in cultivated Brassica varies [55,56]. Notably, according to Dubrow et al. [57] the nonpathogenic *X. campestris* pv. *campestris* weed isolate 5053 lacked the T3SS and associated type III effector (T3E) genes, suggesting that loss of these key pathogenicity determinants may contribute to the nonpathogenic lifestyle of some weed-associated isolates. In contrast, other isolates recovered from weeds were pathogenic, indicating that weed-associated *X. campestris* populations are heterogeneous in their pathogenicity and virulence-associated genomic features [57].

## 5. Detection and Identification

**Figure 3:**
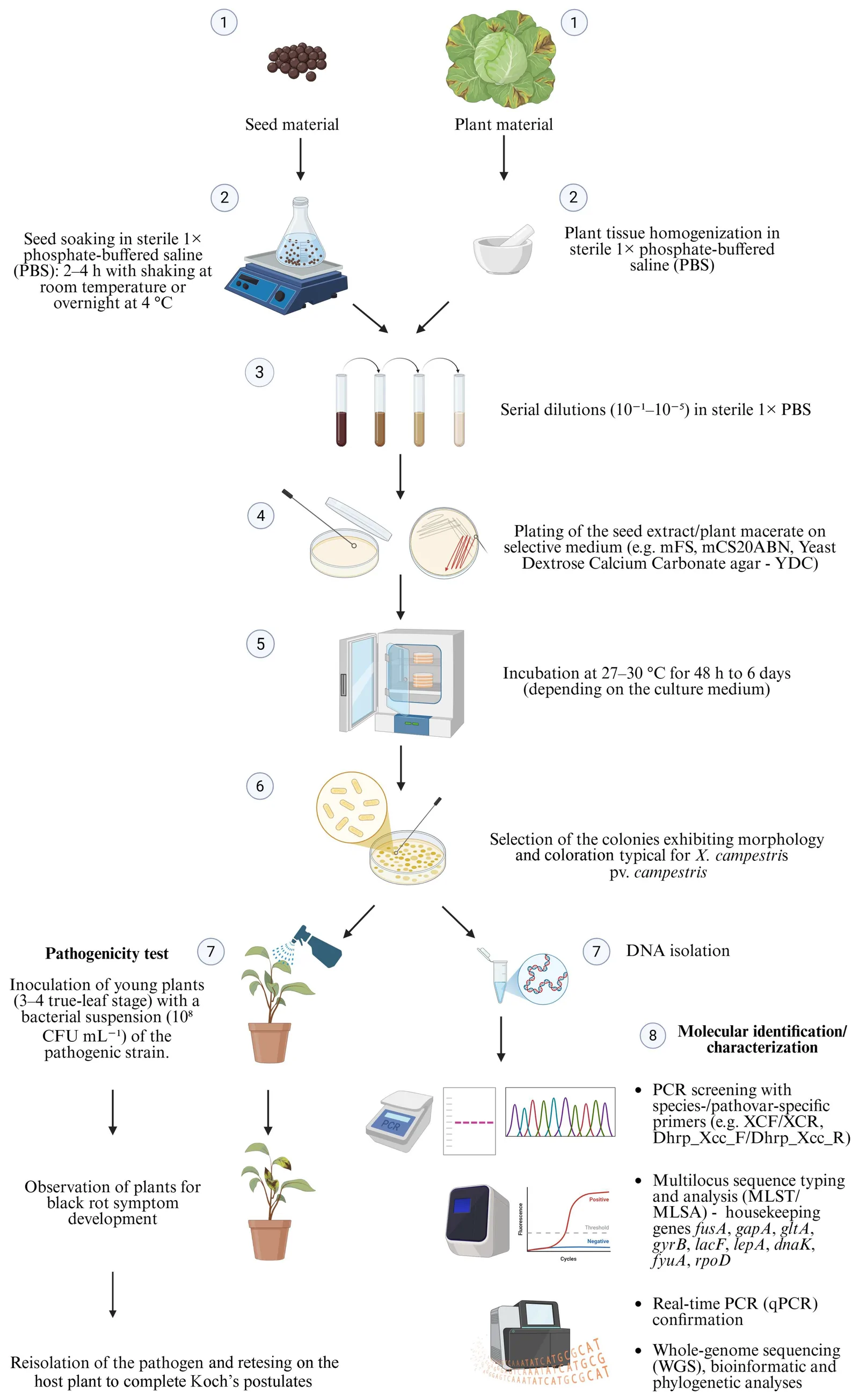
Diagnostic workflow for *X. campestris* pv. *campestris* from crucifers seed and plant material (created in https://BioRender.com); graphical representation of the information presented in this section.

Considering the importance of seed as a primary source of inoculum for *X. campestris* pv. *campestris* and its role in the long-distance dissemination of the pathogen, numerous diagnostic methods have been developed for *Brassica* seed health-testing and phytosanitary control. Bacterial cultivation is a time-consuming process (3–10 days), particularly for slow-growing bacteria such as *X. campestris* pv. *campestris*; therefore, the development of PCR-based methods has been of great importance for rapid pathogen detection [58,59]. Accordingly, the International Seed Testing Association (ISTA) has, since 2013, recommended the use of DNA markers as an alternative to pathogenicity tests to facilitate the discrimination of *X. campestris* pv. *campestris* from closely related *Xanthomonas* pathovars [60]. The use of species-specific primers for the routine detection of phytopathogenic bacteria has also been recommended in most EPPO protocols [61,62]. According to the latest ISTA protocol [63], the semi-selective media mFS and mCS20ABN are used for the isolation of seedborne pathogens (*X. campestris* pv. *campestris* and *X. campestris* pv. *raphani*) from *Brassica* seeds. Their selectivity is primarily achieved through the incorporation of selective agents, namely nystatin, cephalexin, and trimethoprim in mFS, and nystatin, neomycin sulphate, and bacitracin in mCS20ABN, whereas soluble starch facilitates the differential recognition of suspect *Xanthomonas* colonies through characteristic zones of starch hydrolysis [63]. After five days of incubation at 28 °C typical colonies of *X. campestris* pv. *campestris* should be surrounded by zones of starch hydrolysis on these media. Additionally, the use of Yeast extract-dextrose-calcium carbonate (YDC) medium is recommended for subculture, on which strains of *X. campestris* pv. *campestris* form typical pale yellow, mucoid, colonies after three days of incubation at 28 °C (Figure 3) [30,31,63]. The ISTA protocol includes an optional seed-extract qPCR pre-screening step to rapidly identify negative seed lots before culture-based confirmation on selective medium. However, PCR or qPCR may detect DNA from viable as well as non-viable bacterial cells. Therefore, a positive molecular result should be confirmed by plating on semi-selective media to demonstrate the presence of viable bacteria, followed by appropriate identification and pathogenicity testing when required. Presumptive colonies may be confirmed using PCR with species- and pathovar-specific primers targeting specific genomic regions, providing high sensitivity and specificity for seed health testing. Specific PCR amplification of the highly conserved gene *hrp*, which is responsible for the hypersensitive response and pathogenicity and plays a crucial role in establishing the interaction between the phytopathogenic bacterium and its host, enables rapid preliminary detection of *X. campestris* pv. *campestris* in plant or seed samples. Several primer pairs have been proposed for the specific identification of *X. campestris* pv. *campestris*, including XCF/XCR, Dhrp_Xcc_F/Dhrp_Xcc_R, and Zup2309/Zup2310, all of which amplify different regions of the *hrpF* gene [64,65,66] (Figure 3). Rubel et al. [67] designed three pairs of pathovar-specific primers, namely ‘Xcc_48F/R’, ‘Xcc_53F/R’ and ‘Xcc_79F/R’, which amplify 855, 930 and 1573 bp fragments, respectively, exclusively from *X. campestris* pv. *campestris*, thereby enabling its differentiation from other *Xanthomonas* pathovars. In addition to species- and pathovar-specific primers for pathogen detection, increasing attention has recently been directed toward the development of type-specific primers capable of distinguishing different *X. campestris* pv. *campestris* races without the need to use differential *Brassica* cultivars. To date, race-specific primers have been reported for the detection of races 1–9 of *X. campestris* pv. *campestris* [68,69,70,71,72,73,74].

Spectral scanning, particularly hyperspectral imaging combined with artificial intelligence, represents a promising approach for the early detection of seedborne pathogens [75]. This approach may have potential for the early detection of *X. campestris* pv. *campestris* in infected seeds, although its application to this pathogen has not yet been specifically demonstrated.

*X. campestris* is a highly genetically heterogeneous species that has undergone substantial genetic selection over time [76]. Genetic diversity in *X. campestris* may also be influenced by extrachromosomal elements such as plasmids, which are present in a subset of strains and may contribute to genome plasticity and adaptation [77]. Recent findings suggest that the loss of CRISPR-Cas immunity in the evolution of *X. campestris* pv. *campestris* may have facilitated plasmid acquisition and increased genome plasticity, potentially promoting the acquisition of virulence-associated traits [78]. Due to this genetic heterogeneity, various molecular methods have been used to characterize *X. campestris* populations and assess their genetic diversity, including DNA fingerprinting and genotyping methods such as restriction fragment length polymorphism (RFLP), random amplified polymorphic DNA (RAPD), amplified fragment length polymorphism (AFLP), and repetitive extragenic palindromic PCR (rep-PCR), as well as multilocus sequence typing/analysis (MLST/MLSA) and genome sequencing [55,57,76,79,80,81,82,83]. Sequencing of the 16S rRNA gene in *Xanthomonas* spp. Produces sequences that are either completely homogeneous or insufficiently divergent to allow the discrimination of closely related species within this genus. The minimal nucleotide differences that can be detected in 16S rRNA gene sequences represent random mutations that have been retained in the genome under no (or limited) selection pressure [84]. To gain better insight into the genetic structure of the large number of pathogenic and non-pathogenic bacteria, the MLST approach began to be extensively applied for their identification and characterization in 1998 [85]. This approach is based on the sequencing of at least four (usually seven or eight) genes encoding essential metabolic functions [85,86]. Mutations in these genes are considered to be under neutral selection, making them more likely to provide an accurate representation of the phylogeny of bacterial strains [87]. Two MLST schemes, based on different combinations of conserved genes, have been proposed for the identification and genetic characterization of bacteria belonging to the genus *Xanthomonas*: (i) Young et al. [79]: *dnaK* (DnaK molecular chaperone), *fyuA* (TonB-dependent receptor), *gyrB* (DNA gyrase B), and *rpoD* (RNA polymerase sigma factor RpoD); and (ii) Almeida et al. [86]: *fusA* (elongation factor G), *gapA* (glyceraldehyde-3-phosphate dehydrogenase A), *gltA* (citrate synthase), *gyrB*, *lacF* (phosphotransferase system lactose-specific EIIA component), and *lepA* (elongation factor 4) (Figure 3). In addition to the application of MLSA for the interpretation of MLST analysis results, Feil et al. [88] proposed the use of the goeBURST (globally optimized implementation of the eBURST algorithm) algorithm, which is based on the determination of allelic profiles for each of the sequenced (most commonly seven) conserved genes and their assignment to a specific sequence type (ST), i.e., genotype, based on the combination of allelic profiles. The application of this algorithm enables the identification of ancestral genotypes and the determination of evolutionary relationships within a species. Allele-based analysis has been applied in several studies investigating the diversity of *X. campestris* pv. *campestris*, but using different combinations of conserved genes [30,31,32,76,89,90,91].

With the development of next-generation sequencing (NGS) technologies, whole-genome sequencing (WGS) has emerged as a powerful tool for the high-resolution identification and characterization of bacterial pathogens, including *X. campestris* pv. *campestris* (Figure 3). Unlike single- or multi-locus approaches, WGS provides genome-wide information, enabling accurate assessment of genetic relatedness, phylogenetic relationships, and population structure among strains. Comparative genomic analyses based on WGS data can be used to identify genetic determinants associated with pathogenicity and virulence, as well as genes involved in host adaptation and other relevant phenotypic traits. WGS has also facilitated the identification of single nucleotide polymorphisms (SNPs), insertions and deletions (indels), and other genomic variations that can be used for high-resolution discrimination of closely related *X. campestris* pv. *campestris* strains. Therefore, WGS represents a valuable complement to conventional molecular approaches such as MLST/MLSA particularly for detailed investigation of the genetic diversity, evolutionary relationships of *X. campestris* pv. *campestris* populations [57,77].

Next to the genetic characterization, pathogenicity testing represents the crucial step for the identification after pathogen isolation (Figure 3). According to the ISTA protocol [63], pathogenicity is tested in susceptible *Brassica* seedlings (e.g., cabbage cv. Wirosa, known as susceptible to all races of *X. campestris* pv. *campestris*) at the 3–4 true-leaf stage by inoculating the major leaf veins of young leaves with suspect isolates. In addition to this type of inoculation, spraying of plants with the inoculum of the pathogenic strain (1 × 10^8^ CFU/mL) is another largely exploited method of pathogen application [6,30,31]. Plants are assessed for the development of characteristic ‘V-shaped’ chlorotic to necrotic lesions and blackening of the leaf veins, which generally develop within 10–14 days. The appearance of these symptoms, in comparison with appropriate positive and negative controls, confirms the pathogenicity of the isolate and supports its identification as *X. campestris* pv. *campestris*. After pathogenicity has been confirmed, the final step in pathogen identification is the re-isolation of the pathogen from symptomatic inoculated plants, thereby completing Koch’s postulates (Figure 3).

## 6. Conventional Control

The cultural management approach implies several different strategies aimed at reducing primarily inoculum and limiting pathogen survival, thereby allowing the natural reduction in the pathogen population over time [92]. These practices include crop rotation with plants from other families (non-host of *X. campestris* pv. *campestris*), growing plants in fields that have been free of cruciferous crops for at least two years, use of pathogen-free planting material (seeds or transplants), and soil draining and drying [28]. According to the work of da Silva et al. [92] the ideal crops recommended for rotating systems with crucifers are carrot, chive, maize, sorghum, spinach, sunn hemp, sunflower, and zucchini, due to the low survival of *X. campestris* pv. *campestris* in their phyllosphere and rhizosphere.

Physical treatment involves treating seeds with hot water (45–55 °C) or ultraviolet light (different UV-C doses), seed disinfection with 3% hydrogen peroxide, ensuring a significant reduction in bacterial populations [93,94]. However, despite its effectiveness in reducing or eliminating seed-borne *X. campestris* pv. *campestris*, hot-water treatment may adversely affect seed germination and vigor, particularly when excessive temperatures or prolonged exposure times result in seed damage [28]. According to Sanna et al. [94] the most efficient method for seed disinfection against *X. campestri*s pv. *campestris*, without compromising the germination process, is the immersion of small quantities of seeds enclosed in cotton bags, in a hydrogen peroxide solution for 15 min at room temperature (25–27 °C), followed by drying for 2 h. Nanoparticles have also been successfully used for the control of black rot by damaging cellular structures and interfering with DNA, RNA, and protein synthesis, ultimately resulting in bacterial cell death [95,96,97,98].

Chemical control is commonly used as a part of integrated management strategies for the reduction in black rot disease of crucifers. Among these, copper-containing products, with copper oxychloride and copper hydroxide have been the most commonly exploited [52]. However, their excessive, prolonged and widespread application resulted in the spread of copper-resistance genes among saprophytic and plant-pathogenic bacteria, resulting in decreased efficacy [99]. Hsiao et al. [100] demonstrated that the *copA* gene, encoding a multicopper oxidase, plays a crucial role in copper tolerance and contributes to the virulence of *X. campestris* pv. *campestris*, highlighting its role in adaptation to copper-based bactericides. In countries where the use of antibiotics in plant protection is permitted, antibiotics such as streptomycin, oxytetracycline, quinolones, and kasugamycin are used to control *Xanthomonas* spp.; however, despite their high efficacy during the initial stages of application, their intensive use rapidly leads to the selection of antibiotic-resistant strains [101,102,103,104]. Resistance in xanthomonads can arise via chromosomal mutations (e.g., *rpsL*-associated streptomycin resistance) or the horizontal acquisition of resistance determinants (e.g., *strAB* and *tet* genes), mainly as a result of the repeated or preventive application [104]. Consequently, long-term and often inappropriate and excessive use of synthetic pesticides and antibiotics has contributed to environmental contamination, while also negatively affecting beneficial organisms and increasing pesticide residues in food.

Among the available disease management strategies, host resistance is considered one of the most effective and economically viable approaches for the long-term control of black rot, complementing or potentially reducing the need for chemical control. According to Vicente & Holub [19], the major resistance gene for black rot is rare in cultivated *B. oleracea*, making it challenging to identify highly resistant genotypes within this species. In contrast, valuable resistance sources have been identified in other *Brassica* species, particularly *B. rapa* and *B. carinata*, as well as in the wild relative *Arabidopsis thaliana*. These species thus represent crucial genetic resources for breeding cabbage cultivars resistant to black rot [105,106]. In a more recent study, Greer et al. [107] showed that the distribution of resistance in *B. oleracea* (genome CC, 2n = 18) and *B. napus* (genome AACC, 2n = 38) differs depending on the *X. campestris* pv. *campestris* race. While resistance to races 1 and 4 remains limited in *B. oleracea*, this species represents an important source of resistance to races 5 and 6. The gene-for-gene model, proposed by Vicente et al. [17], may facilitate the development of successful breeding programs for resistance to *X. campestris* pv. *campestris*. The model describes the interactions between *X. campestris* pv. *campestris* races and different *Brassica* cultivars [*B. oleracea* (Wirosa F1 and Miracle F1), *B. rapa* (Just Right Hybrid Turnip and Seven Top Turnip), *B. napus* (Cobra line 14R), *B. carinata* (PI 199947), and *B. juncea* (Florida Broad Leaf Mustard)] and is based on the interaction of at least four corresponding pairs of genes, namely avirulence genes (A1–A4) and race-specific resistance genes (R1–R4). According to the proposed model, the resistance of the Cobra oilseed rape line 14R is explained by the interaction between the R4 resistance gene (derived from the A genome of *B. rapa*) and the A4 avirulence gene. The model further suggests that the R1 and R4 genes play a key role in resistance to the most economically important *X. campestris* pv. *campestris* races, namely races 1 and 4. Ignatov et al. [108] also reported race-specific resistance in different oilseed rape genotypes, with most of the tested genotypes exhibiting a high level of resistance to at least one of the three *X. campestris* pv. *campestris* races evaluated (races 1, 4, and 5). In a subsequent study, Vicente et al. [105] identified three oilseed rape lines—one winter line derived from the cultivar Cobra and two spring lines, CrGC5 and N-o-1—as resistant to *X. campestris* pv. *campestris* race 4, demonstrating that this resistance is controlled by a single dominant gene, Xca4. Likewise, Taylor et al. [106] found that several winter and spring oilseed rape cultivars were resistant to *X. campestris* pv. *campestris* race 4, although spring cultivars tended to exhibit only partial resistance. The same authors also observed variable levels of resistance to races 3 and 5 among *B. napus* and *B. rapa* genotypes. The most effective strategy for developing durable resistance is to combine major resistance genes against the most common pathogen races (R1 and R4) with non-race-specific genes that confer broad-spectrum quantitative resistance [19]. When it comes to the resistance of *B. oleracea* to *X. campestris* pv. *campestris*, Vega-Álvarez et al. [109] recently demonstrated a pronounced diurnal variation in disease resistance, showing that inoculation at the end of the photoperiod (PM) consistently resulted in lower disease severity than inoculation in the morning (AM). Recent advances in CRISPR/Cas9-mediated editing of the BoMLO12 gene have successfully produced novel *B. oleracea* germplasms with enhanced broad-spectrum resistance and significantly improved yield performance. Such findings of Zhang et al. [110] represent a foundation for breeding *B. oleracea* varieties with increased disease resistance and productivity. In addition to race-specific resistance, vascular resistance in the stem can prevent the systemic spread of *X. campestris* pv. *campestris* within the plant, representing an important alternative mechanism of defense in *B. oleracea* [111]. Ignatov et al. [111] demonstrated that the stem vascular system of several *Brassica oleracea* accessions showed resistance to *X. campestris* pv. *campestris*, which prevented systemic spread of the pathogen through the stem vascular system. This resistance was race-nonspecific and represents an alternative mechanism of plant defense that can limit systemic infection by black rot.

## 7. Biological Control

As the world’s population grows exponentially and environmental concerns intensify, the need for introducing more sustainable forms of production has become crucial to mitigate climate change and protect biodiversity [112]. In response to these challenges, scientists shifted their attention towards developing alternatives to synthetic agrochemicals, which resulted in the development of more environmentally friendly biological control agents and biopesticides [113]. IPM represents one such approach combining several strategies to effectively control plant diseases while minimizing the excessive use of chemical pesticides and their undesirable effects on the environment and human health [114]. Within the IPM framework, sustainable disease management encompasses both biological control based on beneficial organisms and the application of naturally derived products, including plant-based biochemicals [115]. Biological control implies the use of a range of ‘green’ management practices that include use of different biochemicals, macroorganisms, as well as microorganisms and their derivatives in disease control [115,116].

In addition to microbial biocontrol agents, plant-derived products have attracted increasing attention as environmentally friendly alternatives for plant disease management. Among these, essential oils have emerged as promising botanical biopesticides due to their broad-spectrum antimicrobial activity and biodegradability [117]. These metabolites play important roles in plant defense by activating host resistance mechanisms and by directly affecting the structure and physiological functions of pathogenic microorganisms [117,118]. Essential oils can be classified into four principal chemical groups: terpenes, terpenoids, phenylpropanoids, and a diverse group of other compounds derived from fatty acids, lactones, glycosides, or sulfur- and nitrogen-containing metabolites. Although terpenes such as p-cymene and limonene generally exhibit limited antimicrobial activity, terpenoids (e.g., thymol, carvacrol, linalool, geraniol, and menthol) and phenylpropanoids (e.g., eugenol, cinnamaldehyde, and vanillin) are considered the main contributors to the antimicrobial properties of essential oils due to their effectiveness against a broad range of microorganisms [119]. Numerous studies have demonstrated that essential oils exhibit broad-spectrum antimicrobial activity against various plant pathogens, including *X. campestris* pv. *campestris* [119,120,121,122]. Although numerous essential oils have demonstrated strong antibacterial activity against *X. campestris* pv. *campestris* under in vitro conditions, studies evaluating their in vivo efficacy (greenhouse or field conditions) remain scarce. Consequently, their practical application for black rot management is still limited, largely due to challenges associated with formulation and field persistence due to their volatility and poor environmental stability, as well as potential non-target effects on beneficial microorganisms present on the plant surface [123,124].

To express their beneficial properties, biocontrol agents must be adapted to both abiotic (environmental) and biotic (host-specific) factors. Adaptation is often overlooked in biocontrol research; however, the poor performance of biocontrol agents under field conditions is most commonly a consequence of inadequate adaptation rather than insufficient biocontrol activity. As specified by Zeriouh et al. [125] colonization and persistence on the plant surface or in the soil are considered major challenges for the application of bacterial biocontrol agents. This is of particular importance for the control of pathogens infecting aerial plant parts, where biocontrol agents are typically applied to the phyllosphere and are therefore exposed to substantial fluctuations in temperature, humidity, and nutrient availability. Therefore, the application of autochthonous phyllosphere strains as biocontrol agents is considered an ecologically efficient strategy for the control of foliar pathogens given that they occupy the same ecological niche, thereby overcoming the key problem of adaptation to environmental conditions [6,126]. Similarly, microorganisms that inhabit the rhizosphere are considered ideal for use in biocontrol since the rhizosphere represents the first line of defence of plant roots against soil-borne pathogens [127]. For seedborne pathogens such as *X. campestris* pv. *campestris*, the use of seed-associated or seed-applied biocontrol agents may provide an additional opportunity to target the pathogen at the seed stage, where successful colonization and persistence are also critical for biocontrol efficacy.

Among microbial biocontrol agents, bacteriophages have gained increasing attention as biocontrol agents for the management of bacterial plant diseases, including black rot caused by *X. campestris* pv. *campestris*. As bacterial natural enemies, bacteriophages are found wherever their hosts occur and lyse them with high specificity, often infecting only a few strains of a particular bacterial species [128]. According to Beber et al. [128], among the tailed phages infecting *X. campestris* pv. *campestris*, most of the morphologically characterized phages exhibit myoviral morphology, in contrast to previous observations within the genus *Xanthomonas*, where siphoviral phages were reported to be the predominant morphotype among tailed phages. The same authors specified that only three filamentous *X. campestris* pv. *campestris* phages have been morphologically described to date. According to the current International Committee on Taxonomy of Viruses (ICTV) summarized by Beber et al. [128], phages infecting *X. campestris* pv. *campestris* have been assigned to the genera *Salvovirus*, *Carpasinavirus*, *Foxunavirus*, *Foxquatrovirus*, *Eisenstarkvirus*, *Rockefellervirus*, and *Lophivirus*, while several phages remain unclassified. In a study conducted by Papaianni et al. [129], the combination of *X. campestris* pv. *campestris-*specific phage Xccϕ1, 6-pentyl-α-pyrone (6PP), a secondary metabolite produced by *Trichoderma atroviride* strain P1, and mineral hydroxyapatite (HA), collectively referred to as the ϕHA6PP complex, was shown to interfere with the gene pathways involved in the formation of biofilm of *X. campestris* pv. *campestris*, thereby contributing to pathogen control. As specified in the previous example, antimicrobial peptides produced by fungi are receiving increasing attention as potential biocontrol agents. Among these, peptaibol trichogin GA IV, produced by another *Trichoderma* species, *T. Longibrachiatum*, exhibited antimicrobial activity against *X. campestris* pv. *campestris*, both in vitro and in vivo on cauliflower plants [130]. However, research on fungal biocontrol agents for black rot management is limited compared with studies focusing on bacteriophages, beneficial bacteria, and their antimicrobial metabolites, possibly due to their lower activity [28].

Bacterial-based bactericides have been considered among the most promising alternatives to chemical pesticides, offering the advantage of leaving no hazardous residual effects on the treated crops and posing less risk to the environment and end consumers [131,132]. They may protect plants from pathogens either (i) directly (interaction biocontrol agent–pathogen) by competing with pathogenic microbes for space and nutrients and/or by producing secondary metabolites with antimicrobial activity [e.g., volatile organic compounds (VOCs), lipopeptides, and antibiotics], or (ii) indirectly (interaction biocontrol agent–host plant) by induction of systemic resistance in plants [131,133,134]. To overcome the challenges associated with colonization and persistence on plant surfaces and in the soil, bacteria employ various mechanisms, including flagellar motility and biofilm formation, which are important for the successful establishment and persistence of biocontrol agents [135]. Among bacterial biocontrol agents, strains belonging to the genera *Bacillus* (e.g., *B. amyloliquefaciens*, *B. megaterium*, *B. subtilis*, *B. pumilus*, *B. velezensis*) and *Pseudomonas* (e.g., *P. orientalis*, *P. aeruginosa*) have been most extensively investigated for the suppression of black rot caused by *X. campestris* pv. *campestris*, although promising results have also been reported for members of the genera *Burkholderia*, *Cupriavidus*, *Leuconostoc*, *Paenibacillus*, and *Streptomyces* [6,9,10,32,136,137,138,139,140,141,142,143,144]. Particular attention has recently been given to the use of plant-associated bacteria as biocontrol agents, as it is increasingly recognized that microorganisms that have co-evolved with their host plants are well adapted to the plant environment and can provide multiple benefits. Their long-term association with a particular host enables them not only to compete effectively with plant pathogens but also to promote plant growth and improve plant resilience. Consequently, indigenous plant-associated microorganisms are considered particularly promising candidates for the development of selective and sustainable biocontrol strategies [145].

Considering the broad spectrum antimicrobial activity of the *Bacillus* species, their capacity to colonize plant tissues, and production of numerous bioactive metabolites, they have emerged as the most extensively studied bacterial biocontrol agents for the management of black rot. Additionally, *Bacillus* spp. represent one of the most widely studied and commercially exploited groups of bacterial biocontrol agents, with numerous strains developed and registered for plant protection. Their diverse antimicrobial activities and strong potential for formulation and practical application further support their prioritization in this review. Accordingly, the following section will provide an overview of the current state of knowledge on the application of *Bacillus* spp.

## 8. The Role of *Bacillus* spp. and Their Secondary Metabolites in the Biological Control

Bacteria from the genus *Bacillus* are ubiquitous, both in terrestrial and water environments. They have an ability to multiply fast and are resistant to adverse environmental conditions due to the ability to develop stress-resistant dormant spores [146]. Given the mentioned characteristics, *Bacillus* species are considered ideal candidates for the formulation of efficient, stable, and long-lasting biopesticide products [147]. Additionally, *Bacillus* species are able to produce a plethora of bioactive secondary metabolites with exceptional antimicrobial traits, including three main classes—bacteriocins, lantibiotics, and miscellaneous antibiotics [148]. Genome sequencing of different *Bacillus* species revealed that even 8% of their genome is dedicated to synthesizing antibiotics [149]. The most common and earliest documented natural products from *Bacillus* are cyclic lipopeptides. Among them, the greatest attention is being given to families of surfactins, iturins, fengycins, and kurstakins [150,151]. Lipopeptides produced by *Bacillus* spp. are involved not only in direct antagonistic activity but also in bacterial attachment to plant surfaces and biofilm formation, which protects microbial cells from adverse environmental conditions. Certain antibiotics, particularly lipopeptides, also facilitate bacterial motility, most likely by altering the viscosity of colonized surfaces [152]. In addition, surfactins and fengycins have recently been identified as elicitors of induced systemic resistance (ISR) in the host plant [125]. In the study by Leclère et al. [153] the lipopeptides surfactin and mycosubtilin produced by *B. subtilis* were shown to promote the spreading of *B. subtilis* cells over surfaces through their surface-active properties, by reducing surface tension and increasing surface wetting. Surfactins produced by *B. subtilis* have also been shown to induce membrane permeabilization [154]. Due to its amphiphilic nature, surfactin can interact with the lipid membranes of other microorganisms, including pathogens. Exposure to surfactin can also alter membrane phospholipid composition, which may disrupt membrane integrity and contribute to its antimicrobial activity [154,155]. Fengycins and iturins have been shown to exhibit primarily strong antifungal activity, with comparatively weaker antibacterial and antiviral effects, whereas surfactins display the most pronounced antibacterial and antiviral activities [156,157]. Next to the cyclic lipopeptides, gaseous metabolites (denominated as VOCs: alcohols, alkanes, acids, ketones, etc.) are also being intensively studied as potential antimicrobials [158,159]. However, there are differences in the number of produced bioactive compounds, as well as in their concentration, depending on the *Bacillus* species or even within different strains of the same species, which are mostly influenced by cultivation conditions [159]. During mass production, these metabolites are released into the growth media and can be used in the biocontrol product with or without the living cells of the antagonists [152]. Also, the established ability of a particular strain to produce some bioactive compound(s) in vitro does not necessarily correlate with their in situ antagonism. Understanding the mode of action is crucial for achieving optimum disease control [152]. Some species within the genus also express plant growth-promoting properties due to the ability to enhance plant nitrogen fixation, phytohormone production (e.g., gibberellic acid and indole-3-acetic acid), nutrient solubilization, and 1-aminocyclopropane-1-carboxylate (ACC) deaminase activity [160,161].

Several recent studies have investigated the biocontrol potential of indigenous strains isolated from cruciferous plants infected with *X. campestris* pv. *campestris*. Such strains are considered promising because they are already adapted to the ecological conditions of agricultural soils’ and crucifers’ microbiome, which may facilitate their establishment and persistence after application, while also enabling more effective competition with the pathogen. Phyllosphere-associated strains are primarily exposed to environmental stresses on aerial plant surfaces and may be particularly relevant for direct protection of leaves, whereas rhizosphere-associated strains are adapted to the root environment and may provide protection through rhizosphere colonization, competition with soilborne pathogens, antibiosis, and induction of plant defenses. Thus, niche adaptation may contribute to the ability of *Bacillus* strains to establish and persist in the target environment and may ultimately affect their biocontrol efficacy. In the recent study, Stanojević et al. [6] reported that four autochthonous *B. velezensis* strains (coded as P-FC 55, RD-FC 88, R-FC 102, and R-FC 114) obtained from the phyllosphere and rhizosphere of cabbage cv. Futoški (with or without black rot symptoms) exhibited pronounced in vitro, as well as in vivo antagonistic activity against *X. campestris* pv. *campestris*, especially when applied as preventive foliar treatments. Even though strains showed genetic potential for the production of surfactins, iturins (except for the strain R-FC 114), and bacillomycin D, ultra-high-performance liquid chromatography coupled to quadrupole time-of-flight tandem mass spectrometry (UHPLC–QtoF MS) revealed only the production of surfactins (C12–C17) under certain growing conditions. In the same study, gas chromatography/mass spectrometry (GC/MS) analysis of ethyl acetate extracts identified a wide range of compounds with potential antimicrobial activity, such as 2,3-butanediol, urea, succinic acid, thymine, phenylalanine, and 9H-purin-6-ol. Similarly, Jelušić et al. [10] demonstrated that *B. velezensis* strain X5-2 and *B. megaterium* strain X6-3, isolated from the phyllosphere of oilseed rape, effectively controlled bacterial blight-like disease caused by *X. campestris* pv. *campestris*. Among the compounds with potential antimicrobial activity, strain X5-2 showed genetic potential for the production of surfactins, kurstakins, and bacillomycin D synthetase, as well as the genes of the iturin operon, while *B. megaterium* X6-3 only had genes for surfactins and kurstakin synthetase. From the cyclic lipopeptides that may have contributed to the antimicrobial activity, high-pressure liquid chromatography system interfaced with a qTOF mass spectrometer (HPLC-ESI-qTOF/MS) analysis of ethyl acetate extracts of these strains indicated the production of compounds corresponding to kurstakins (C11, C12, and C13), surfactins (C12–C15), iturins (C15 and C16), and bacillomycin D (C14 and C15) by *B. velezensis* X5-2, while only surfactins with a fatty acid chain length of C12–C16 were detected in *B. megaterium* X6-3. The VOCs 2,3-butanediol, methoxymethane, and heneicosane were exclusively present in the ethyl acetate extract of *B. velezensis* X5-2. Conversely, GC–MS analysis showed that the ethyl acetate extract of *B. megaterium* X6-3 was characterized by relatively high peak areas corresponding to the carboxylic acids 2-methylbutanoic acid, 3-methylbutanoic acid, and 2-methylpropanoic acid, as well as acetic acid, compared with the other isolates evaluated in the study [10]. VOCs, 2-methylbutanoic acid, 3-methylbutanoic acid, and 2-methylpropanoic acid have also been also previously reported to possess both antifungal and antibacterial activities [162,163]. In addition, two pyrazines, 3-propan-2-yl-2,3,6,7,8,8a-hexahydropyrrolo [1,2-a]pyrazine-1,4-dione and 3-benzyl-2,3,6,7,8,8a-hexahydropyrrolo [1,2-a]pyrazine-1,4-dione, together with the alkanes dodecane and tetradecane, detected in the extracts of *B. megaterium* X6-3, were also identified in the dichloromethane extract of *B. megaterium* BmBP17, which exhibited antibacterial activity against the phytopathogens *Xanthomonas axonopodis* pv. *punicae* and *Ralstonia solanacearum* [164]. The pyrazine derivative 3-benzyl-2,3,6,7,8,8a-hexahydropyrrolo [1,2-a]pyrazine-1,4-dione was earlier reported in the ethyl acetate extract of *B. velezensis* strain PEA1, where it exhibited antifungal and antiviral activities [165]. The mentioned 2,3-butanediol has been previously shown to induce systemic resistance in plants [166]. Moreover, it was demonstrated to promote stomatal closure in *A. thaliana* and *Nicotiana benthamiana* Domin., thereby preventing the entry of foliar pathogens into plant tissues [167]. Such a mode of action could be especially important for vascular pathogens like *X. campestris* pv. *campestris*, whose main entry points are the stomata.

Metabolomic profiling of the antagonistic *B. velezensis* strain FZB42, isolated from cabbage [168], suggested that the combined effect of surfactin, fengycin, and bacillomycin D contributes to its antagonistic activity against *X. campestris* pv. *campestris* under in vitro conditions. Grady et al. [166] identified the lipopeptides [Leu7] surfactin C14 and [Leu7] surfactin C15 in *B. velezensis* strain 9D-6, which exhibited biocontrol activity against a range of plant pathogens. Among the eight surfactin isoforms detected in *Bacillus* spp., surfactins C14 and C15 were shown to possess the greatest potential for disease suppression [167]. Lipopeptide families, including kurstakins, surfactins, iturins, and bacillomycins, were detected in the extract of *B. velezensis* strain NST6, with the anti-staphylococcal activity being attributed to bacillomycin D C15 [169]. According to Mishra & Arora [9], *B. thuringiensis* strain SE, originating from the rhizosphere soil of *B. campestris* showed high efficacy in reducing black rot disease under greenhouse conditions when applied either as a foliar spray or as a combination of seed soaking and soil drenching. In the same study, two antibacterial factors corresponding to autolysins (β-N-acetylglucosaminidase) and AHL-lactonases were identified in the biocontrol strain SE using nuclear magnetic resonance (NMR) spectroscopy. Additionally, the combination of the two autochthonous strains *B. thuringiensis* strain SE and *Pseudomonas aeruginosa* KA1 was significantly more effective in black rot control when the mode of application was a combination of seed soaking and soil drenching [9]. However, when it comes to biocontrol agents such as *P. aeruginosa* and *B. cereus*, which belong to Biosafety Group: RG-2 (associated with human disease, rarely serious; preventive or therapeutic interventions often available), their potential use should be carefully evaluated in terms of biosafety and regulatory requirements. Although individual strains may show effective biocontrol activity, their potential risks to human health and the environment may limit their suitability for practical application, and the use of safer, well-characterized strains or alternative species should therefore be prioritized. Another potential antagonistic mechanism involves interference with the diffusible signal factor (DSF) signaling system, involved in regulation of virulence factors in *X. campestris* pv. *campestris*. Newman et al. [170] identified bacterial strains belonging to the genera *Bacillus*, *Paenibacillus*, *Microbacterium*, *Staphylococcus*, and *Pseudomonas* that were capable of rapidly degrading DSF, thereby disrupting DSF-mediated induction of virulence factors in *X. campestris* pv. *campestris*. In a recent study, Kim et al. [11] demonstrated significant antibacterial activity of the soil-derived *B. velezensis* strain 21–128 against *X. campestris* pv. *campestris*, both in vitro and in vivo, and identified surfactins C13–C16 as major lipopeptide compounds associated with this activity. Another study analyzing 51 indigenous *Bacillus* strains originating from *Brassica* seeds and plants highlighted the potential of strains identified as *B. amyloliquefaciens*, *B. subtilis* and *B. pumilus* as the most effective in reducing black rot incidence in vivo [141]. Their metabolic profiling revealed the production of several secondary metabolites, including surfactin, iturin, bacillomycin, and azalomycin F, suggesting that these compounds may contribute to the biocontrol potential of *Bacillus* spp. against *X. campestris* pv. *campestris*.

Overall, the studies demonstrate the considerable potential of *Bacillus* spp. as biological control agents against *X. campestris* pv. *campestris*, both in vitro and in vivo. Future research should focus on translating this accumulated knowledge into practical applications through the selection of robust isolates, elucidation of their modes of action, and development of effective formulations and application strategies.

## 9. Challenges and Future Perspectives

Despite advances in pathogen detection and identification, *X. campestris* pv. *campestris* remains a serious threat to *Brassica* cultivation worldwide. Under ongoing climate change and the continuous development of new *Brassica* cultivars, the evolutionary dynamics of *X. campestris* pv. *campestris* is expected to continue, with the potential emergence of new pathogen races and populations with altered virulence profiles that may compromise the effectiveness of currently available disease management strategies. In this context, the exploitation of indigenous antagonistic strains, especially different *Bacillus* species, represents a promising approach, as microorganisms originating from the same host and ecological niche as the pathogen may be better adapted to colonize and persist in that environment, potentially providing a competitive advantage over introduced biocontrol strains. However, a *Bacillus* strain effective against a particular crop is not necessarily restricted to the region from which it was isolated and may potentially be applied to the same crop grown in different geographical regions, provided that it remains effective under the prevailing environmental and cultivation conditions. Future research should therefore focus on understanding the mode of action underlying the antimicrobial activity of these candidate strains, while further improving their formulation, application strategies, and performance under field conditions. Such efforts could facilitate the development of more stable and effective biological control products that can be integrated into IPM programs for the sustainable management of black rot.

## Data Availability

No new data were created or analyzed in this study. Data sharing is not applicable to this article.
